# Competing Biases in Mental Arithmetic: When Division Is More and Multiplication Is Less

**DOI:** 10.3389/fnhum.2017.00037

**Published:** 2017-02-01

**Authors:** Samuel Shaki, Martin H. Fischer

**Affiliations:** ^1^Department of Behavioral Sciences, Ariel UniversityAriel, Israel; ^2^Division of Cognitive Science, University of PotsdamPotsdam, Germany

**Keywords:** heuristics and biases, numerical cognition, mental arithmetic, mental number line, operational momentum

## Abstract

Mental arithmetic exhibits various biases. Among those is a tendency to overestimate addition and to underestimate subtraction outcomes. Does such “operational momentum” (OM) also affect multiplication and division? Twenty-six adults produced lines whose lengths corresponded to the correct outcomes of multiplication and division problems shown in symbolic format. We found a reliable tendency to over-estimate division outcomes, i.e., reverse OM. We suggest that anchoring on the first operand (a tendency to use this number as a reference for further quantitative reasoning) contributes to cognitive biases in mental arithmetic.

## Competing Biases in Mental Arithmetic: When Division Is More and Multiplication Is Less

Even in educated adults, understanding quantities is contaminated by systematic biases. This was previously shown in simple arithmetic tasks where they overestimate addition and underestimate subtraction outcomes, both with symbolic and with non-symbolic operands (McCrink et al., 2007; Knops et al., 2009, 2014). For example, when presented with a horizontal line flanked by 0 and 10 on both sides, adults indicate the result of “4 + 2” further rightward than the result of “8–2” (Pinhas and Fischer, 2008; see also Pinhas et al., 2014, 2015). These mental arithmetic biases are called “operational momentum” (OM) effects.

Explanations for OM can be classified into non-spatial vs. spatial accounts, depending on whether they invoke spatial-numerical associations. A first non-spatial account of OM assumes that addition and subtraction are semantically associated with “more” or “less”, respectively (McCrink and Wynn, 2009); this “more-or-less heuristic” assumes that we overestimate addition and underestimate subtraction outcomes due to superficial thinking, regardless of number format or response mode. A second non-spatial account of OM assumes that we calculate, throughout life, with logarithmically compressed number representations, resulting in over- or underestimations of outcomes (see Chen and Verguts, 2012; Knops et al., 2014). This is the “compression account” of OM (e.g., McCrink et al., 2007).

In contrast, spatial accounts of OM postulate cognitive operations on a “mental number line” (MNL), a spatially oriented representation of number concepts in ascending order (for review see Fischer and Shaki, 2014): The “attentional-shift account” suggests that OM originates from movements of attention along the MNL, causing “overshoots” in the direction associated with the arithmetic operation, namely leftward (towards smaller numbers) for subtraction and rightward (towards larger numbers) for addition (Knops et al., 2013, 2014; see also Klein et al., 2014). Alternatively, the “spatial-competition account” postulates that the spatial activations induced by the operands (i.e., single numbers), the operator (i.e., plus or minus signs) and the computed outcome (Pinhas and Fischer, 2008; Pinhas et al., 2014, 2015) together induce OM. This account acknowledges multiple sources of OM but has not yet been sufficiently elaborated to determine the relative weights of these contributions to overall OM.

Given the evidence for OM in addition and subtraction, similar biases should exist in multiplication and division. Examining these predictions is important because we expect fundamental principles of cognition to generalize across cognitive operations. Moreover, comparing across tasks can refine our understanding of any given cognitive mechanism, such as its range of applicability. For example, it is puzzling to see larger OM in zero- compared to non-zero problems (e.g., 3 + 0 vs. 2 + 1) although the second operand “zero” requires no movements along the MNL (see Pinhas and Fischer, 2008; Pinhas et al., 2015).

We derived the following predictions: first, the more-or-less heuristic generalizes to state “if addition is more, multiplication is much more” and “if subtraction is less, division is much less” (for similar reasoning, see the “multiplication makes bigger, division makes smaller” (MMBDMS) heuristic of Katz and Knops, 2014). This reasoning captures the fact that multiplication can be characterized as repeated addition, although this is usually only regarded as typical of less advanced calculators. Similarly, the size of attention shifts should scale with the operator, inducing larger biases for multiplication than addition and larger biases for division than subtraction. Next, according to the compression account, larger numbers are more compressed than small numbers. As a result, the dividend is more compressed than the divisor, leading to underestimation of the outcomes of division problems. As both operands in multiplication problems are much smaller than of the dividend, their mental representations are less compressed, leading to higher outcome estimation than in division, and strong OM is predicted. Finally, in the absence of empirical evidence about spatial associations of these operators, and without using zero as an operand, spatial competition predicts larger OM for multiplication than division.

A recent study (Katz and Knops, 2014) attempted to measure, for the first time, OM in multiplication and division. Adult participants were presented with two operands, one on each side of the operator, and both in either symbolic or non-symbolic format. This was followed by a choice display containing five different possible solutions in the same format. The authors found OM with non-symbolic dot patterns: a bias to select larger than correct outcomes in multiplication and smaller than correct outcomes in division. However, there was no OM with symbolic operands. The authors explained this null result with the fact that response choices were all larger than the operands for multiplication, and smaller than the operands for division. Therefore, the proposed MMBDMS heuristic could not be applied. Alternatively, the lack of OM for symbolic numbers may reflect the fact that participants had to select 1 of 5 visually presented numbers, thus triggering overlearned fact knowledge that can be directly retrieved from memory (e.g., Campbell, 1987; Ashcraft, 1992). For a similar failure to find OM with symbolic notation using this method, see Knops et al. (2009). However, the relatively low accuracy levels for symbolic multiplication (87%) and division (74%) speak against this alternative explanation.

Here we adopt an improved method to re-assess the presence of OM with symbolic numbers in multiplication and division. Specifically, in contrast to Katz and Knops (2014) discrete symbolic number choices, we present a line whose length must match the outcome of a multiplication or division problem (for details, see below; for previous applications of this production method, see Shaki et al., 2015). This production task should be more sensitive to the presence of OM in multiplication and division because it provides a direct measure of the outcome of both operations while its non-verbal nature reduces the probability that participants draw on overlearned multiplication and division knowledge.

## Materials and Methods

### Participants

Twenty-six students (19 females, mean age 22.3 years, range 19–28 years, four left-handed) from Ariel University participated in one 45-min session for course credit. All participants reported normal or corrected-to-normal vision and were naïve about the purpose of the experiment.

### Stimuli and Apparatus

**Figure 1:**
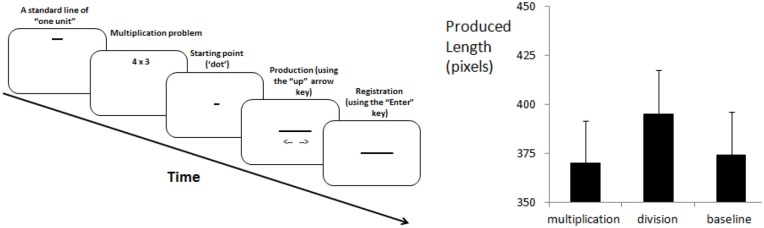
**Methods (left panel) and main results (right panel) of the experiment (error bars = 1 SEM)**.

The method is visualized in Figure 1 (left panel). Six two-digit numbers (12, 18, 21, 24, 27 and 28), six multiplication problems (4 × 3; 6 × 3; 7 × 3; 8 × 3; 9 × 3 and 7 × 4) and six division problems (48:4; 36:2; 63:3; 48:2; 54:2 and 112:4) constituted the stimulus set. These problems were taken from Katz and Knops (2014; Table 1) and their two-digit outcomes served as a baseline. Black stimuli were shown on white background (Times New Roman, bold, 30 points). Horizontal lines were three pixels tall and appeared black-on-white on a 19-inch display with 1280 × 1024 pixels resolution (landscape orientation). Each pixel measured 0.25 mm and participants sat approximately 50 cm from the display. The presentation of instructions and stimuli, event timing and response recording were controlled by in-house software. Responses were made using a standard keyboard placed flat on the table with response keys centered under the display.

### Design

We used the production method of Shaki et al. (2015) which removes horizontal spatial biases from participants’ responses (see p. 472 for a more detailed argument). In the beginning of the experiment, two standards, “one unit” (25 pixels) and “forty units” (1000 pixels), were presented on white A4 paper (8.3 × 11.7 inches) in landscape orientation. Participants’ task was to produce the line length matching either the magnitude of the 2-digit number or the result of an arithmetic problem (multiplication or division) based on these standards. Participants produced lines with the “Up” and “Down” arrow keys.

For one group of participants, the line (or “starting-point”) initially was a “dot” (2 pixels wide) and for the other group the “starting-point” was a line 1000 pixels wide. Each stimulus, either a number or a problem, was repeated six times per block, resulting in 108 trials for each participant. Stimulus order was randomized within a block and participants were assigned randomly to the two “starting-point” conditions.

### Procedure

Upon arrival, participants signed a consent form before looking at the standards. They performed the task without standards being visible. On each trial, one randomly selected stimulus was presented 200 ms after participants pressed an arrow key. The stimulus was shown for 600 ms; then the display turned blank until participants pressed one of the mid-sagittally aligned (near or far) arrow keys to display the starting point for responding. Each button press adjusted line lengths by 2 pixels (↑ for longer, ↓ for shorter lines); continuous pressing adjusted line length at 30 Hz. Both increasing and decreasing length adjustments were permitted. When participants were satisfied they pressed the “Enter” key to register their response; this started the next trial without feedback.

## Results

An analysis of variance (ANOVA) evaluated effects of three stimulus types (two-digit numbers, multiplication, division) and six target numbers (12, 18, 21, 24, 27, 28) as within-subjects factors and two starting points (dot, line) as between-subject factor on line-length productions. The main effect of stimulus type was significant, *F*_(2,48)_ = 26.44, MSE = 1066, *p* < 0.001, partial *η*^2^ = 0.524. Contrary to the OM prediction, participants produced longer lines for results of division (395 pixels) than for results of multiplication (370 pixels) or two-digit numbers (374 pixels; see right panel of Figure 1). *Post hoc* comparisons with 0.05-level with Bonferroni correction indicated that division results were longer than either multiplication results or two-digit numbers. However, multiplication results did not differ from two-digit numbers.

The effect of target number was also significant, *F*_(5,120)_ = 114.29, MSE = 4557, *p* < 0.001, partial *η*^2^ = 0.826: as instructed, participants produced longer lines for larger target numbers. The interaction stimulus type X target number, *F*_(10,240)_ = 2.22, MSE = 1726, *p* = 0.017, partial *η*^2^ = 0.085, reflected larger increments in division compared to the other conditions, specifically for largest outcomes. Average line lengths did not reliably differ between starting-point groups, *F*_(1,24)_ < 1, with means of 388 and 371 pixels for the “line” and “dot” starting conditions, respectively, and also did not interact with any factors, all *F’s* < 1.42, *p* > 0.17.

## Discussion

Comparing both operation outcomes to the baseline reveals that multiplication outcomes were not different from the baseline. However, participants produced longer lines in division than in the baseline condition. Thus, contrary to prediction, we found a reverse OM effect: participants produced longer lines to reflect division outcomes compared to multiplication outcomes. Reverse OM was previously reported by Knops et al. (2013) in non-symbolic addition and subtraction, perhaps reflecting lack of arithmetic knowledge in the children tested. It was also found in a spatial pointing task with adults by (Pinhas et al. (2015), see also Klein et al., 2014), due to reversal of the visually presented number interval. Importantly, none of the OM accounts can explain the reverse OM we found when comparing multiplication and division.

In order to meet this challenge, we propose the presence of two competing heuristics and biases in mental arithmetic:

(1) A “more-or-less” heuristic—building on the suggestion of McCrink and Wynn (2009) for addition and subtraction, Katz and Knops (2014) argued that OM can also originate from the intuition that “multiplication makes bigger, division makes smaller” (MMBDMS). This intuition is gradually acquired through daily life, where multiplications create larger outcomes and divisions create smaller outcomes, hence biasing our estimation or acceptance of multiplication and division outcomes.
(2) An anchoring bias—for identical arithmetic outcomes, the first operand is (on average) larger in division than in multiplication. Hence, division involves initial activation of larger number concepts compared to multiplication (see Tversky and Kahneman, 1974, for a demonstration of anchoring within multiplication). Anchoring is present early in development (Smith, 1999) and is a powerful and unconscious cognitive mechanism (Kahneman, 2011).

These two components of OM can explain the present results as well as several previous observations. Reverse OM, indicated by longer lines for division than multiplication outcomes, reflects strong anchoring on the large first operands in division, overruling the weaker “more-or-less” heuristic. The lack of difference between multiplication and baseline outcomes reflects (a) stronger anchoring in baseline trials due to our controlled outcomes; (b) the counteracting influence of the “more-or-less” heuristic that is only relevant in arithmetic operations and not for single numbers.

Anchoring is an explanation for reverse OM in division when compared to multiplication. Why has it not been noticed in subtraction problems where the first operand is also bigger? Indeed, we think that anchoring sheds a fresh light on the previous puzzle of larger OM for zero compared to non-zero problems (Pinhas and Fischer, 2008; Pinhas et al., 2015): specifically, the computation of OM for zero problems is based on comparing problems with identical first operands (e.g., 3 + 0 vs. 3–0), thus equating the anchoring effect. In contrast, computing OM for non-zero problems with controlled outcomes (e.g., 2 + 1 vs. 4–1) dilutes OM in the second example because its first operand is larger and its associated anchoring bias counteracts the expected bias towards smaller numbers and thus the overall OM.

Why has anchoring not yet been acknowledged as a relevant factor in mental arithmetic? This could be due to a prevalence of problems with similar small-number concepts that are not sufficiently different from each other to reveal the influence of anchoring. Alternatively, anchoring has been overlooked because mentally calculating with symbolic notation, especially in the small number range, taps retrieval processes and thus leaves little uncertainty that enables anchoring to emerge, at least with traditional methods.

We briefly address two methodological concerns: first, anchoring is also reflected in perceptual biases, e.g., when producing lines from a small or large starting point, respectively (see Jewell and McCourt, 2000; Shaki et al., 2015). The absence of this perceptual anchoring in the present results probably reflects the between-group manipulation of the starting point condition and helps us to compare the results between groups. Second, both groups were Hebrew speaking healthy adults who read arithmetic problems from left to right; hence, the first operand is their anchor and reading direction for text is an irrelevant concern here (see also Fischer and Shaki, 2016).

Considering the proposed interplay of the “more-or-less” heuristic and the anchoring bias also explains the results of Katz and Knops (2014) who found non-symbolic OM: the initially available anchoring was probably diluted by the presentation of multiple dot patterns for OM asse, 1974, p. 1131), “… adjustment from an anchor (…) is usually employed in numerical prediction when a relevant value is available.”

An open issue at this point is the lack of complete reversal of OM in subtraction when compared to the present division outcomes. We assume that the use of a production task in the present study removed contributions to OM from other spatial biases, such as attention shifts or sign-space associations (see above). These factors contribute to the magnitude of OM but were absent here, thus leaving only two competing players in the OM game and supporting our assumption of the strength of anchoring. Future studies are directed at determining the relative weights of the various components of OM, ideally by investigating all arithmetic operations in a single study. Possible cognitive benefits of OM need to be investigated in future studies. In this endeavor it might also be useful to apply neuroimaging techniques (e.g., Naseer and Hong, 2015) to assess brain regions involved in the different components of OM.

## Ethics Statement

This study was carried out in accordance with the recommendations of “Faculty Ethics committee of Ariel University” with written informed consent from all subjects. All subjects gave written informed consent in accordance with the Declaration of Helsinki. The protocol was approved by the “Faculty Ethics committee of Ariel University”.

## Author Contributions

SS and MHF equally contributed to the manuscript.

## Conflict of Interest Statement

The authors declare that the research was conducted in the absence of any commercial or financial relationships that could be construed as a potential conflict of interest.
